# Interfacial Electronic Effects in Co@N-Doped Carbon Shells Heterojunction Catalyst for Semi-Hydrogenation of Phenylacetylene

**DOI:** 10.3390/nano11112776

**Published:** 2021-10-20

**Authors:** Yuan Huang, Haoting Yan, Chenyang Zhang, Yize Wang, Qinhong Wei, Renkun Zhang

**Affiliations:** Department of Chemical Engineering, School of Petrochemical Engineering & Environment, Zhejiang Ocean University, Zhoushan 316022, China; huangyuan9930@163.com (Y.H.); a1193930739@163.com (H.Y.); zcy646387783@126.com (C.Z.); wyz20010110603870@163.com (Y.W.)

**Keywords:** cobalt catalyst, N-doped carbon, Mott-Schottky effect, catalytic hydrogenation, phenylacetylene

## Abstract

Metal-supported catalyst with high activity and relatively simple preparation method is given priority to industrial production. In this work, this study reported an easily accessible synthesis strategy to prepare Mott-Schottky-type N-doped carbon encapsulated metallic Co (Co@N_p+g_C) catalyst by high-temperature pyrolysis method in which carbon nitride (g-C_3_N_4_) and dopamine were used as support and nitrogen source. The prepared Co@N_p+g_C presented a Mott-Schottky effect; that is, a strong electronic interaction of metallic Co and N-doped carbon shell was constructed to lead to the generation of Mott-Schottky contact. The metallic Co, due to high work function as compared to that of N-doped carbon, transferred electrons to the N-doped outer shell, forming a new contact interface. In this interface area, the positive and negative charges were redistributed, and the catalytic hydrogenation mainly occurred in the area of active charges. The Co@N_p+g_C catalyst showed excellent catalytic activity in the hydrogenation of phenylacetylene to styrene, and the selectivity of styrene reached 82.4%, much higher than those of reference catalysts. The reason for the promoted semi-hydrogenation of phenylacetylene was attributed to the electron transfer of metallic Co, as it was caused by N doping on carbon.

## 1. Introduction

The selective hydrogenation of alkynes to alkenes is one of the most important chemical reactions in the chemical industry [1,2]. Among the produced alkenes, styrene is a very important chemical raw material and is used as a monomer for the production of polystyrene, resin, and styrene butadiene rubber [3]. In the current ethylene cracking unit, styrene as a by-product is unavoidably produced, along with a small amount of phenylacetylene. In styrene polymerization, the blended phenylacetylene could lead to poison and even deactivation of catalysts [4,5], seriously influencing the polymerization of styrene. To remove the phenylacetylene, hydrogenation of phenylacetylene over metal-based catalysts is viewed as an effective and feasible avenue [6]. Different from the physical separation that is of considerable difficulty because of their similar molecular structure, the catalytic hydrogenation could transform phenylacetylene to styrene directly. However, the catalytic hydrogenation of carbon-carbon triple bonds in phenylacetylene molecule to carbon-carbon double bonds encounters a big challenge because the carbon-carbon triple bond, especially in a terminal position, is subject to over-hydrogenation and then form ethylbenzene [7]. Therefore, the selective hydrogenation of phenylacetylene to styrene is in urgent need of being promoted [6,8].

With regard to the semi-hydrogenation of alkynes to styrenes, it is usually carried out on a typical Lindlar’s catalyst (Pd-CaCO_3_-PbO) [9]. However, the intermediate species alkenes easily evolved to generate alkanes in the pre-desorption process. Despite the presence of the Lindlar’s catalyst, the alkenes are unavoidably formed due to the over-hydrogenation of alkynes. Currently, more efforts have been devoted to developing highly active metal-based catalysts for catalyzing semi-hydrogenation of phenylacetylene [10,11]. In particular, precious metal catalysts showed excellent catalytic hydrogenation activity in phenylacetylene hydrogenation. Yu et al. reported that the prepared Pd/PCFs catalyst for the hydrogenation of phenylacetylene and the conversion of phenylacetylene was up to 93%. Unfortunately, the selectivity of styrene was only 62% [4]. In addition, Pt-based catalysts have also been studied for many hydrogenation reactions widely [12,13,14,15,16]. Carbon nanotube-supported Pt catalyst (CN@Pt/CNTs) reported by Xia et al. was employed for hydrogenation of phenylacetylene [17]. Both phenylacetylene conversion and styrene selectivity almost attained 100%. Even though noble metal-based catalysts posed some limitations toward largle-scale applications because of relatively high cost. Considering the economic rationality in the application, the transition-metal catalysts, such as Ni, Co, and Fe, have been investigated in the catalytic hydrogenation of alkynes to alkenes [18]. On the whole, these transition-metal catalysts showed satisfactory catalytic performances [19,20,21,22,23,24,25].

For supported transition-metal catalysts, ZrO_2_, Al_2_O_3_, SiO_2_, zeolite [26], etc [27]. are commonly used as supports in that they are earth-abundant and widely available metal oxides [28]. Moreover, carbon, which possesses high surface area, abundant surface species, and unique electronic properties, is a well-known support material as well as a catalyst in various hydrogenation reactions [29,30,31,32]. It is worth mentioning that compared with pure carbon material, doped and/or modified carbon materials are more suitable as catalyst supports, contributing due to the enhanced electronic structure to the potential catalytic events [33,34]. Recently, nitrogen-doped carbon has been studied and developed for heterogeneous catalytic reactions [35]. Nitrogen doping can significantly increase the electron density of states (DOS) of inert carbon carriers [18], reduce the electronic spin density of metal nanoparticles, and improve the oxidation resistance of metal nanoparticles [36]. Furthermore, the electronic interaction between nitrogen-doped carbon and active metal is strengthened, effectively enhancing the stability and promoting the catalytic activity of the metal catalyst [37,38,39,40]. From the perspective of solid-state physics, the N-doped carbon and loaded metal are regarded as semiconductor and conductor, respectively. When intimate contact occurs between them, a new metal-semiconductor interface (called Schottky barrier) is constructed, which leads to the electron transfer between semiconductors and metal. As a result, the contact interface of the heterostructure plays an important role in catalytic reactions. Inspired by the above elaborations, we strive to take a simple route to construct a N-doped carbon-supported metal Mott-Schottky-type catalyst that possesses high catalytic activity in selective hydrogenation of phenylacetylene [41,42,43,44,45].

In this work, Mott-Schottky-type N-doped carbon encapsulated metallic cobalt catalyst with core-shell structure was prepared by a high-temperature pyrolysis strategy for phenylacetylene hydrogenation. This encapsulation structure of Co@N_p+g_C formed a strong electronic interaction between metal Co and N-doped carbon outer shell, generating a Mott-Schottky effect. Metal Co, a conductor which possesses a lower work function than N-doped carbon (P-type semiconductor), transferred electrons to N-doped carbon. The Mott-Schottky barrier was therefore formed in their interface, where the positive charges were enriched in the side of metallic Co. The positively charged metallic Co exhibited suitable catalytic activity in phenylacetylene hydrogenation, and high selectivity of styrene was obtained. The reason for the promoted semi-hydrogenation of phenylacetylene was mainly attributed to the electric structure regulation of metallic Co.

## 2. Materials and Methods

### 2.1. Materials

Cobalt nitrate hexahydrate (Co(NO_3_)_2_•6H_2_O), melamine (C_3_H_6_N_6_), dopamine hydrochloride (C_8_H_11_NO_2_•HCl), D_(+)_-Glucose and commercial silicon dioxide were purchased from Aladdin (Shanghai, China). Tris (hydroxymethyl) aminomethane (C4H11NO3), ethanol absolute (C6H6O), phenylacetylene (C8H8) were provided by Sinopharm Chemical Reagent Co., Ltd., Shanghai, China. All the above reagents were used directly without any other treatment.

### 2.2. Preparation of Catalyst

Preparation of Co@N_p+g_C catalyst: before catalyst preparation, g-C_3_N_4_ was first prepared by directly calcining melamine. The procedure of preparation is as follows: A certain amount of melamine was transferred to a porcelain crucible and then heated in a muffle furnace from room temperature to 520 °C at a heating rate of 3 °C min^−1^ and kept at this temperature for 3 h. After cooling the crucible to room temperature, g-C_3_N_4_ was obtained.

Tris (hydroxymethyl) aminomethane (0.242 g), dopamine hydrochloride (0.8 g), and g-C_3_N_4_ (0.8 g) were added into deionized water (200 mL) with rigorous stirring for 30 min. Then, cobalt nitrate hexahydrate (0.3 g) was added to the mixture with stirring for another 24 h. The resulting suspension was collected by filter, washed with deionized water, and then dried in an oven at 120 °C overnight. Finally, the dark gray precursor was pyrolyzed in a tubular furnace at 700–900 °C in a nitrogen atmosphere for 2 h to obtain N-doped graphene shell encapsulated metal Co catalyst (Co@N_p+g_C). Moreover, without the addition of g-C_3_N_4_ during the preparation process, the prepared catalyst was designated as Co@N_p_C. In addition to the Co@N_p+g_C catalyst, the Ni@N_p+g_C and Fe@N_p+g_C catalysts were prepared for contrast analysis. 

For comparison, N-doped carbon-supported Co catalyst without core-shell structure (Co/NC) was also prepared. The preparation process of Co/NC catalyst is almost the same as that of Co@N_p+g_C catalyst except for the replacement of dopamine hydrochloride with D_(+)_-Glucose. As well known, catalyst support plays an important role in the catalytic activity of catalysts. To highlight the difference of supports in catalytic reactions, SiO_2_ supported Co catalyst (Co/SiO_2_) was prepared. The preparation procedure adopted the impregnation method, followed by drying and calcination (500 °C for 2 h). Before the reaction, the Co/SiO_2_ was reduced to 500 °C for 2 h.

All these prepared catalysts possessed a similar loading amount of about 5 wt% as detected by inductively coupled plasma mass spectrometry (ICP-MS).

### 2.3. Characterization

The power X-ray diffraction (XRD) patterns of samples were recorded on a Rigaku MiniflexII X-ray diffractometer (JEOL, Tokyo, Japan) with Cu-Kα radiation at an operating condition of 40 kV and 40 mA at a scanning speed of 2 °/min. The transmission electron microscopy (TEM) images, high-resolution transmission electron microscopy (HRTEM) images, and scanning transmission electron microscopy images with high angle angular, dark field (STEM-HAADF) were observed on JEM-2100F microscopy, JEOL, Tokyo, Japan. The X-ray photoelectron spectra (XPS) of samples were carried out on AXIS ULTRA DLD spectrometer (Kratos, Manchester, UK) with an X-ray source of Al Ka. The ultraviolet photoelectron spectra (UPS) of samples were performed on a Thermo Escalab 250Xi spectrometer (Thermo Fisher Scientific, Waltham, MA, USA) equipped with HeI irradiation (hv = 21.21 eV). The defect degrees of samples were tested by Raman spectrum on a HORIBA Scientific LabRAM HR Evolution spectrometer (HORIBA Jobin Yvon, Longjumeau, France) with a Peltier-cooled CCD detector at a laser wavelength of 514 nm. The loading amounts of metallic Co of samples were evaluated by inductively coupled plasma mass spectrometry (ICP-MS) on an Agilent 7700X spectrometer (Agilent Technologies Inc., Palo Alto, CA, USA). The specific surface area and pore structure of samples were tested by low-temperature N_2_ physical adsorption on a QUADRASORB SI instrument (Quantachrome, FL, USA). Before the test, the samples were purified under vacuum at 200 °C for 2 h.

### 2.4. Catalyst Evaluation

The selective hydrogenation of phenylacetylene was carried out in a 20 mL Teflon-lined autoclave equipped with a magnetic stirrer. In a typical procedure, 30 mg catalyst, 100 µL phenylacetylene, and 6 mL ethanol were placed in the sealed autoclave. The autoclave was purged with pure H_2_ several times and then charged with H_2_ to 2 MPa. The autoclave was heated to 120 °C for a catalytic reaction for 2 h. The obtained products were analyzed by gas chromatography with an Agilent HP-5 commercial capillary column. The conversion of reactant and selectivity of the key product were calculated on the basis of the following equations:

Conversion (%) = {Phenylacetylene feed (mol) − Phenylacetylene residue (mol)} × 100%/Phenylacetylene feed (mol)

Selectivity (%) = Styrene product (mol) × 100%/{Phenylacetylene feed (mol) − Phenylacetylene residue (mol)}

## 3. Results and Discussion

### 3.1. Structure and Surface Morphology of the Co@NC Catalyst

**Figure 1:**
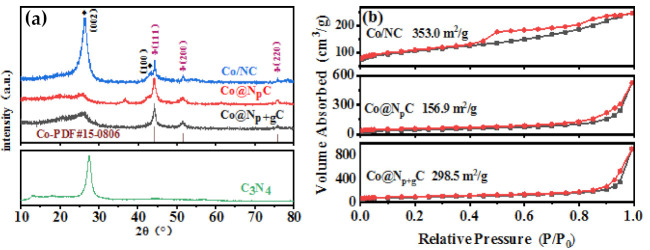

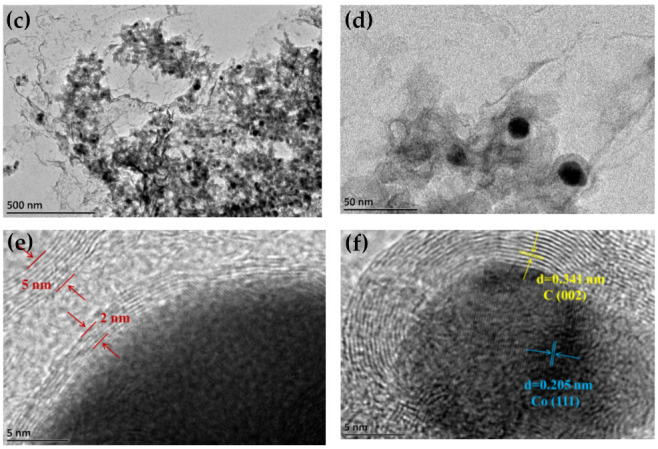
XRD patterns (**a**) and N_2_ adsorption-desorption isotherms (**b**) of all samples. TEM and HRTEM images of the Co@N_p+g_C (**c**–**f**).

XRD profiles of samples are shown in Figure 1a. Two diffraction peaks positioned at 26° and 43° are attributed to the (002) and (100) reflection planes of graphite carbon, respectively [46]. Other diffraction peaks located at 44.2°, 51.5°, and 75.5° were assigned to (111), (200), and (220) planes of metallic Co^0^ [46]. The XRD results indicated that metal Co species were directly reduced to metallic Co during high-temperature pyrolysis. Meanwhile, the average particle size of Co was calculated to be 18 nm by XRD. However, except for the metallic Co peak, the Co@N_p_C displays an extra peak positioned at 36.6°, which is accorded with the CoO species. The N_2_ adsorption-desorption isotherm curves of samples were performed to measure pore structure in Figure 1b. All isotherms exhibit obvious hysteresis loops. This reveals that a large amount of mesopores were fabricated on catalysts. In particular, the Co@N_p+g_C presents an H1-type hysteresis loop, on which two parallel branches rise steeply in the high P/P_0_ pressure area. Such an isotherm declares that the formed mesopores are highly regular. In the phenylacetylene catalytic hydrogenation reaction, molecules containing benzene rings diffused in the mesoporous channels, which is one of the key steps to ensure the completion of catalytic reactions.

The TEM images of the Co@N_p+g_C prepared by high-temperature pyrolysis using g-C_3_N_4_ and polydopamine as carbon source and nitrogen source were filmed in Figure 1c,d. It is clear that the metallic Co particles are focused on around 15–20 nm. To further reveal the microstructure and surface morphology, the HRTEM images of Co@N_p+g_C were also acquired. Figure 1e shows that the Co nanoparticles are encapsulated with a thin carbon shell (thickness at around 2–5 nm). The fringe spacing of the outer carbon shell is 0.35 nm, larger than that of 0.34 nm for pristine graphene, indicating that the increased lattice spacing is attributed to the N doping onto carbon. The outer N-doped graphene carbon shell, along with inner metallic Co nanoparticles, constructed a classical core-shell structure. In Figure 1f, the HRTEM image presents a lattice fringe of 0.205 nm, which corresponds to the (111) crystal face of metallic Co [47]. By HRTEM images, based on the method of polydopamine coating, the N-doped carbon encapsulated metallic Co was readily accomplished upon high-temperature pyrolysis. Meanwhile, carbon acted as a reducing agent to reduce Co species to metallic Co during pyrolysis. It should be mentioned that the N-doped graphene shell did not completely encapsulate around the metallic Co. It possesses distinct crackings in Figure 1e, allowing the reactant molecules to diffuse into the inside and access active metal Co. For the core-shell structure, electronic interaction between metal Co and N-doped graphene shell was bound to be established because the electron transfer inevitably occurred in the interface between them due to the close contact of conductor and semiconductor (N-doped carbon). As a result, the catalytic activity of metal Co was significantly affected so as to enhance the catalytic activity.

**Figure 2:**
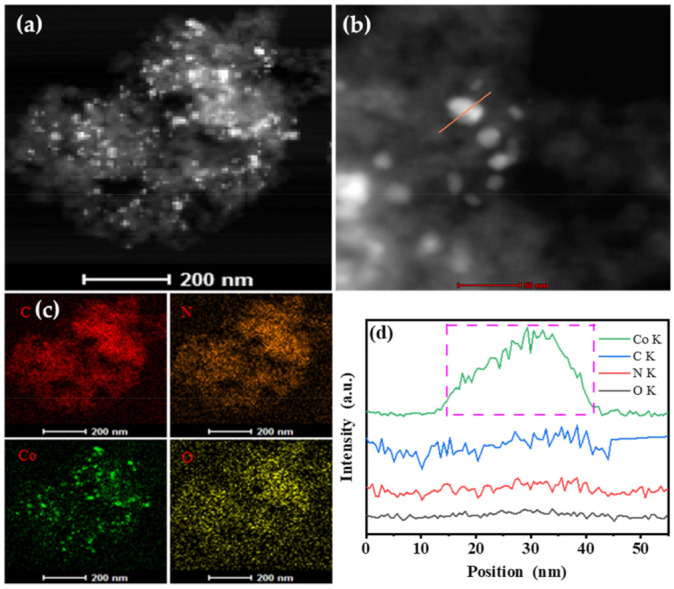
STEM-HAADF images (**a**,**b**), elemental mapping (**c**), and line scanning (**d**) of the Co@N_p+g_C sample.

In order to further reveal the structural information of Co@N_p+g_C, the STEM-HAADF images were taken to provide an adequate illustration. As shown in Figure 2a–c, the high image contrast and element mappings confirm that metal Co NPs were highly dispersed on N-doped carbon, and N dopants spread onto the whole N-doped carbon uniformly. At the same time, EDS (Energy dispersive spectroscopy) line scanning was conducted along the straight direction in Figure 2b. A strong signal of Co K is observed clearly in Figure 2d. However, compared with the Co K signal, the signals of C K, O K, and N K are negligible. The results indicated that Co species existed in the form of a metallic state, not the cobalt carbide and cobalt nitride. HRTEM image, STEM-HAADF image along with EDS line scanning fully verified that the metallic Co NPs encapsulated with N-doped graphene shell could be successfully constructed by high-temperature pyrolysis avenue.

**Figure 3:**
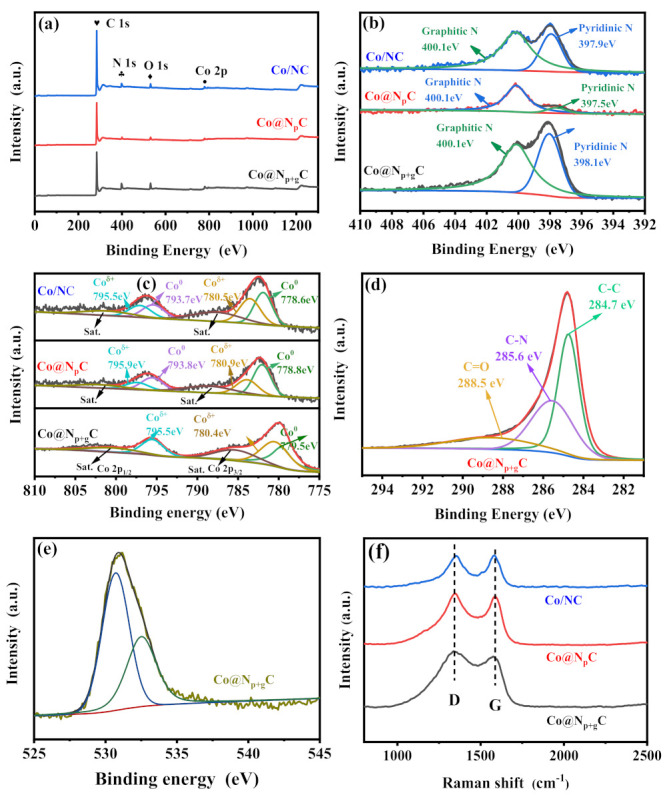
XPS survey (**a**), high-resolution XPS spectra of N 1s regions (**b**), Co 2p regions (**c**), C 1s regions (**d**), and O 1s regions (**e**) for Co@N_p+g_C, Co@N_p_C, and Co/NC samples. Raman spectra (**f**) of Co@N_p+g_C, Co@N_p_C and Co/NC samples.

XPS spectra were employed to analyze the element valence state and local environment of metal Co of different samples. The XPS results (Figure 3a) show that C, N, O, and Co elements were detected in all samples. The Co@N_p+g_C possesses the highest N amount (10.54%) in Table 1, which is consistent with the catalyst preparation. The N 1s spectra of samples were deconvoluted, and there are two N 1s peaks observed on all of the samples in Figure 3b. The peak at around 398 eV belongs to the pyridinic N, and the other one at around 401 eV is indexed to the graphitic N [48,49]. For N-doped carbon materials, it has been widely recognized that the introduction of pyridinic N into carbon skeleton could interact with supported active metal closely and thus improve the catalytic performance. Our previous study clearly showed that the pyridinic N contributed to the electronic interaction with metal Co [18]. Due to the high content of pyridinic N of Co@N_p+g_C, a stronger electronic interaction could be created between metal Co and pyridinic N species. However, the N 1s spectrum of Co@N_p_C exhibits only a graphitic N peak, without the distinct pyridinic N peak. In response to the N 1s spectra, the Co 2p spectra of all samples were investigated to provide a confirmation that metal Co was composed of Co^0^ and Co^δ+^. As shown in Figure 3c, the high-resolution Co 2p spectra were obtained by Gauss fitting. According to the fitting results, the peaks with binding energy at around 779.5 and 794.6 eV are designated to Co 2p_3/2_ and Co 2p_1/2_ of metallic Co^0^. The peaks located at 780.4 and 795.5 eV are assigned to the Co^δ+^, while the two small peaks at 786.8 and 803.9 eV correspond to the satellite peaks [47]. It is obvious that the Co@N_p+g_C possesses more Co^δ+^ species, and the molar ratio of Co^δ+^/Co^0^ accounts for 1.24 in Table 1. The surface oxidized Co species were originated from the electronic interaction with doped N on carbon because the metal Co with low work function transferred electrons to N-doped carbon when they interacted. With the increase in N content, the interaction was further promoted, resulting in more Co^δ+^ species. The electronic interaction between metal Co core and N-doped graphene shell can therefore be established.

The C1s XPS spectra of samples were also fitted by a Gaussian fitting curve. After deconvolution, three signal peaks with binding energy at 284.7, 285.6, and 288.5 eV were retrieved, which are related to C-C, C-N, and C=O species, respectively in Figure 3d. The presence of C-N indicates that N species were incorporated into the carbon skeleton, forming the pyridinic N and graphitic N as corroborated by N 1s spectra. The fitted O 1s XPS spectra display that two oxygen-based species were formed, which were assigned to the adsorbed oxygen species (531.3 eV) and lattice oxygen species (531.3 eV) [50,51] in Figure 3e; however, the peak of lattice oxygen species at 529.5–530 eV, which is related to the formation of CoO_x_, was absent from the Co@NC [52]. Beyond that, Raman spectra exhibit two typical characteristic peaks of carbon material, corresponding to the D band at 1340 cm^−1^ and G band at 1590 cm^−1^, separately in Figure 3f. By calculating the ratio values of I_d_/I_g_ (D band intensity/G band intensity), the I_D_/I_G_ values of Co@N_p+g_C, Co@N_p_C, and Co/NC were calculated to be 1.29, 1.03, and 0.89, respectively, indicating that the Co@N_p+g_C possessed the more structure defects as caused by more N doping and thus the changed energy structure. There is no Raman peak of cobalt-based oxidation species observed at Raman profiles [53]. All of these characterization results proved that metal Co NPs were effectively encapsulated with N-doped graphene shell, existing steadily in the form of metallic Co along with positively charged Co^δ+^ species.

### 3.2. Catalytic Reactions

#### 3.2.1. Selective Hydrogenation of Phenylacetylene

The selective hydrogenation of phenylacetylene to styrene was performed over Co@N_p+g_C as suitable as reference catalysts at 120 °C and 2 MPa H_2_ in an autoclave. The catalytic results are listed in Table 2. A blank control experiment was first carried out, and the result displays that only 1.03% of phenylacetylene was converted (Table 2, Entry 1). When catalysts were added, the Co/NC and Co@N_p_C obtained 79.2% conversion and 99.1% conversion of phenylacetylene (Table 2, Entry 2, 3), respectively. However, it is interesting that there was no styrene detected over Co@N_p_C catalyst; that is, the phenylacetylene was converted to produce ethylbenzene due to over-hydrogenation. Compared to these two catalysts, the Co@N_p+g_C obtained 92.7% conversion of phenylacetylene, along with high styrene selectivity at 82.4% (Table 2, Entry 4). As reaction pressure increases to 4 MPa, the conversion of phenylacetylene reached 98.6%, and the selectivity of styrene slightly reduced to 80.5% (Table 2, Entry 5). With the 1 MPa reaction pressure, the conversion of phenylacetylene maintained 88.7%, but the selectivity of styrene increased from the original 82.4% to 91.2% (Table 2, Entry 6). The increase in reaction pressure boosted the increase in phenylacetylene conversion while reducing the selectivity of styrene owing to the enhanced hydrogenation ability. Similarly, the Co@N_p+g_C-700 and Co@N_p+g_C-900 catalysts (pyrolyzed at 700 °C and 900 °C) also obtained excellent catalytic activity in phenylacetylene hydrogenation as that of Co@N_p+g_C pyrolyzed at 800 °C (Table 2, Entry 7, 8). The similar structure of Co@N_p+g_C catalysts with different pyrolysis temperatures excluded the influence of other factors; however, the activation of N-doped carbon to metal Co presented an obvious effect. Aside from the Co-based Co@Np+gC catalyst, the Ni@N_p+g_C and Fe@N_p+g_C catalysts were comparatively tested for phenylacetylene hydrogenation. The Ni@N_p+g_C yielded low styrene selectivity, in spite of showing 100% conversion of phenylacetylene. On the contrary, the Fe@N_p+g_C achieved high styrene selectivity but low phenylacetylene conversion. To illustrate the effect of support on catalytic activity, the SiO_2_ supported Co catalyst was measured, and the result displays that the Co/SiO_2_ was of strong hydrogenation activity, and the phenylacetylene was completely converted to generate ethylbenzene.

It can be seen from Table 2 that the Co@N_p+g_C catalyst with N-doped graphene shell has higher catalytic performance than that of Co/NC without core-shell structure as well as general supported catalyst Co/SiO_2_. The N-doped graphene shell, which had strong electronic interaction with metal Co due to close contact between them, could be primarily responsible for the enhanced catalytic activity. This deliberately constructed Mott-Schottky-type catalyst aimed to modulate the electronic structure of active metal Co by imposing the N-doped carbon around it. In particular, the electronic regulation of metal Co slowed down the pace of catalytic hydrogenation, reaching a trade-off of phenylacetylene conversion and styrene selectivity. Even if the change of electrical structure has not yet been clarified, it is most likely to happen on the significant change of 3d orbit electrons [54]. From XPS results, the electrons in Co were partially transferred to the outer N-doped carbon shell and thus made Co positively charged. The electronically regulated Co could be beneficial to the generation of chemical bands between the reactant and catalyst as well as the mild catalytic hydrogenation.

#### 3.2.2. Promotion of Mott-Schottky Effect on Catalytic Activity of Co@N_p+g_C Catalyst

With regard to metal-based heterogeneous catalytic reactions, the surface electronic property of metal active sites has a significant impact on catalytic activity. In this work, we used low-priced raw materials to specially fabricate N-doped carbon shell encapsulated metal Co catalyst by high-temperature pyrolysis method. Firstly, the N doping importantly increased the electronic density of states of pure carbon and changed the inherent energy band structure [53,55,56,57]. Especially for sp^2^-hybrid graphene, its low electron density of states makes it inert [56]. N doping could be capable of redistributing the local electrons of carbon materials, improving the reactivity of carbon carriers [58]. With the N doping, the formed N-doped carbon became a P-type semiconductor [53,55], a higher work function compared to metal Co. It is more difficult for electrons to escape from N-doped carbon [53,59]. When N-doped carbon semiconductor contacted with metal Co conductor, the electron transfer occurred from metal Co to N-doped carbon until their Fermi levels reached equilibrium [60]. Therefore, the electron density of metal Co decreased, partially forming positively charged Co^δ+^. In order to make the Mott-Schottky effect more remarkable, the homemade encapsulated structure could fully show the promoting effect of spatial structure. Unlike the generally N-doped carbon-supported Co that the contact interface of N-doped carbon and metal Co is relatively limited, while the N-doped graphene shell presents all-around contact around metal Co (see XPS in Figure 1f). After contact, a charge region was established at the interface between the N-doped carbon and metal Co because of the redistribution of charges, where the positive charges enriched on one side of metal Co and the negative charges concentrated on the side of N-doped carbon. The catalytic hydrogenation occurred at the interface region consisting of charges.

**Figure 4:**
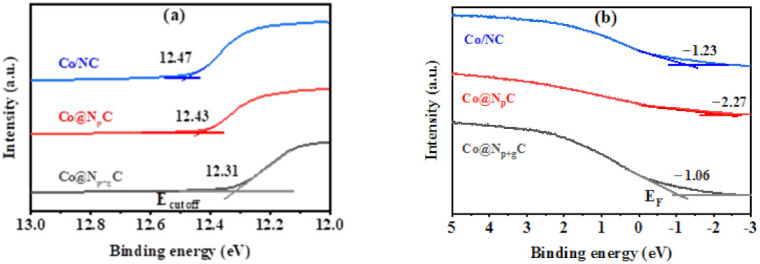

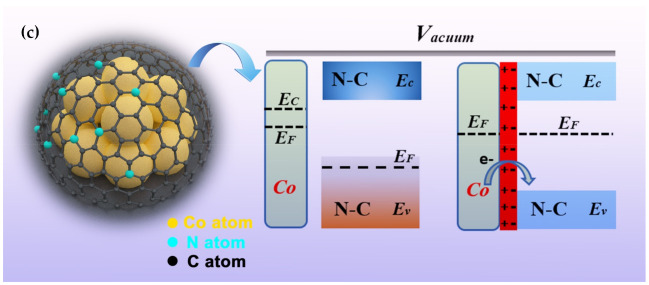
The UPS spectra of samples (**a**,**b**). The work function is based on the photoelectric law W = hν − |E_cutoff_ − E_F_|, where hv is the energy of the incident photon, E_cutoff_ represents the low energy cutoff edge, and E_F_ stands for the Fermi energy level. The mott-Schottky contacting interface of surface metallic Co and N-doped carbon of Co@N_p+g_C samples (**c**).

In order to illustrate the band structure alteration and thus the interfacial electron transfer, UPS spectra were used to calculate the work function (W) of samples based on photoelectric law (W = *hν* − |E_cutoff_ − E_F_|, *hν* = 21.12 eV). As shown in Figure 4a,b, the work function of Co@N_p+g_C, Co@N_p_C, and Co/NC could be calculated by locating secondary electron cutoff edges (E_cutoff_) and Fermi energy (E_f_), them of which being 7.84, 6.51, and 7.51 eV, respectively. Combining the work function as well as the N content from XPS, the higher the N content of the sample is, the greater the work function could be obtained, which was also consistent with our previous research results [18,35]. To describe the contact interface between metal Co and N-doped graphene carbon shells, their energy bands before and after contact are imagined. As displayed in Figure 4c, the metal Co and N-doped graphene shell remain independent state before they contact, and the work function of metal Co is larger than that of N-doped carbon. After contact, Mott-Schottky heterojunction formed was accompanied by an electric field, driving electrons transfer from metal Co to N-doped graphene shell, until their Fermi energy levels reached balance. The result contributed to a new generation of a contact interface with a very thin layer on which the positive and negative charges were redistributed. For the Co@N_p+g_C catalyst with encapsulated core-shell structure, the contact interface of metal and carrier covered the overall outer surface of metal and thus presented a significant Mott-Schottky effect. The catalytic hydrogenation reaction occurred at this interface. By increasing the doping amount of N, the Mott-Schottky contact could be remarkably promoted, finally accomplishing the enhanced catalytic activity.

### 3.3. Catalytic Stability

**Figure 5:**
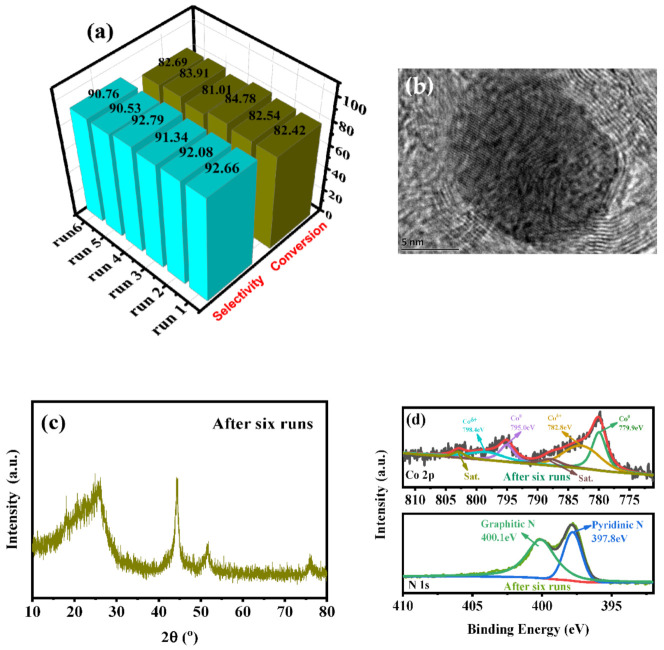
Reaction stability test of the representative Co@N_p+g_C catalyst (**a**); TEM image (**b**), XRD patterns (**c**), Co 2p XPS, and N 1s XPS spectra (**d**) of the spent Co@N_p+g_C after hydrogenation reaction.

In heterogeneous catalytic reactions, the stability of the catalyst is an important indicator. Therefore, the stability of the representative Co@N_p+g_C catalyst was investigated. The stability test shows that there is little change in phenylacetylene conversion and styrene selectivity via six repeated experiments in Figure 5a, proving suitable catalytic stability. At the same time, an HRTEM image of the used Co@N_p+g_C catalyst was filmed. It is clear that the core-shell structure of N-doped graphene shell encapsulated metal Co was intactly preserved after six repeated tests in Figure 5b. With the well-kept core-shell structure, the contact interface was also maintained to ensure the Mott-Schottky effect. This is the reason why the Co@N_p+g_C catalyst maintained excellent catalytic activity and catalytic stability. XRD profile of the used Co@N_p+g_C displays the diffraction peak of metal Co in Figure 5c. The result demonstrated that the stable encapsulated structure is consistent with the HRTEM. In addition, the surface electronic property of the used Co@N_p+g_C was revealed by XPS in Figure 5d. The XPS spectra exhibit similar photoelectron peaks as that of the fresh Co@N_p+g_C. Based on the peak position (at 780 eV), the surface metal Co still mainly exists in the form of Co^δ+^, which resulted from the electronic interaction of metal Co and N-doped graphene shell. According to the characterization results, the Mott-Schottky-type Co@N_p+g_C catalyst retained an extremely stable core-shell structure, by which to initiate the electron transfer and thus gain excellent and stable catalytic activity in the catalytic hydrogenation of phenylacetylene.

## 4. Conclusions

In summary, a Mott-Schottky-type N-doped graphene shell encapsulated metal Co catalyst (Co@Np+gC) was prepared through a high-temperature pyrolysis method using dopamine and carbon nitride as carbon source and nitrogen source. The core-shell structure of Co@N_p+g_C not only stabilized the metal Co NPs but also generated a Mott-Schottky effect. When metallic Co contacted with N-doped carbon shells, the electrons transferred from Co to N-doped carbon shells, which was attributed to the low work function of Co as compared to that of N-doped carbon. The positively charged metallic Co formed in the interface presented excellent catalytic activity for hydrogenation of phenylacetylene to ethylbenzene. The experimental results showed that the Co@N_p+g_C catalyst obtained high styrene selectivity up to 82.4%, while the Co/N_p_C catalyst carried out complete hydrogenation of phenylacetylene to ethylbenzene.

## Figures and Tables

**Table 1:** N content, Co^δ+^ binding energy, and Co^δ+^/Co^0^ molar ratio of Co@N_p+g_C, Co/N_p_C, and Co/NC samples from XPS spectra.

Sample	N wt%	Co^δ+^ Binding Energy	Co^0^ Binding Energy	Co^δ+^/Co^0^ Molar Ratio (%)
Co@N_p+g_C	10.54	780.4 eV/ 795.5 eV	779.5 eV/ 794.6 eV	1.24
Co/NC	6.03	780.9 eV/ 795.9 eV	778.6 eV/ 793.7 eV	0.92
Co/N_p_C	2.96	780.9 eV/ 795.9 eV	778.8 eV/ 793.8 eV	0.60

**Table 2:** Selective hydrogenation of phenylacetylene over different catalysts.

Entry	Catalysts	H_2_ (MPa)	Time (h)	Conv. (%)	Selec. (%)
1	No catalyst	2	2	1.1	83.1
2	Co/NC	2	2	79.2	87.4
3	Co/N_p_C	2	2	99.1	0
4	Co@N_p+g_C	2	2	92.7	82.4
5	Co@N_p+g_C	4	2	98.6	80.5
6	Co@N_p+g_C	1	2	88.7	91.2
7	Co@N_p+g_C-700 ^a^	2	2	98.5	71.1
8	Co@N_p+g_C-900 ^b^	2	2	99.2	70.5
9	Ni@N_p+g_C	2	2	100	47
10	Fe@N_p+g_C	2	2	20.5	82.2
11	Co/SiO_2_	2	2	100	0

Reaction conditions: 30.0 mg of catalyst, 0.5 mmol of phenylacetylene, 6 mL of ethanol, reaction temperature 120 °C. The conversion and selectivity were determined by GC. ^a^ tubular furnace at 700 °C, ^b^ tubular furnace at 900 °C.

## References

[1] Shen Y., Yin K., An C., Xiao Z. (2018). Design of a difunctional Zn-Ti LDHs supported PdAu catalyst for selective hydrogenation of phenylacetylene. Appl. Surf. Sci..

[2] Crespo-Quesada M., Cárdenas-Lizana F., Dessimoz A.-L., Kiwi-Minsker L. (2012). Modern trends in catalyst and process design for alkyne hydrogenations. ACS Catal..

[3] Natarajan T.S., Natarajan K., Bajaj H.C., Tayade R.J. (2013). Enhanced photocatalytic activity of bismuth-doped TiO2 nanotubes under direct sunlight irradiation for deg-radation of Rhodamine B dye. J. Nanoparticle Res..

[4] Yu W., Hou H., Xin Z., Niu S., Xie Y., Ji X., Shao L. (2017). Nanosizing Pd on 3D porous carbon frameworks as effective catalysts for selective phenylacetylene hydrogenation. RSC Adv..

[5] Teschner D., Révay Z., Borsodi J., Hävecker M., Knop-Gericke A., Schlögl R., Milroy D., Jackson S.D., Torres D., Sautet P. (2008). Understanding palladium hydrogenation catalysts: When the nature of the reactive molecule controls the nature of the catalyst active phase. Angew. Chem..

[6] Markov P.V., Mashkovsky I.S., Bragina G.O., Wärnå J., Gerasimov E.Y., Bukhtiyarov V.I., Stakheev A.Y., Murzin D.Y. (2019). Particle size effect in liquid-phase hydrogenation of phenylacetylene over Pd catalysts: Experimental data and theoretical analysis. Chem. Eng. J..

[7] Hub S., Hilaire L., Touroude R. (1988). Hydrogenation of but-1-yne and but-1-ene on palladium catalysts: Particle size effect. Appl. Catal..

[8] Domínguez-Domínguez S., Arias-Pardilla J., Berenguer-Murcia Á., Morallón E., Cazorla-Amorós D. (2008). Electrochemical deposition of platinum nanoparticles on different carbon supports and conducting polymers. J. Appl. Electrochem..

[9] Ulan J.G., Kuo E., Maier W.F., Rai R.S., Thomas G. (1987). Effect of lead acetate in the preparation of the Lindlar catalyst. J. Org. Chem..

[10] Bullock R.M. (2013). Abundant metals give precious hydrogenation performance. Science.

[11] Wang J., Du C., Wei Q., Shen W. (2021). Two-dimensional Pd nanosheets with enhanced catalytic activity for selective hydrogenation of nitrobenzene to aniline. Energy Fuels.

[12] Li C., Shao Z., Pang M., Williams C.T., Liang C. (2012). Carbon nanotubes supported Pt catalysts for phenylacetylene hydrogenation: Effects of oxygen containing surface groups on Pt dispersion and catalytic performance. Catal. Today.

[13] Liu H., Yu Q., Fu H., Wan Y., Qu X., Xu Z., Yin D., Zheng S. (2018). Pt supported on ordered microporous carbon as highly active catalyst for catalytic hydrodeiodination of iodinated X-ray contrast media. Appl. Catal. B Environ..

[14] Wu Q., Zhang B., Zhang C., Meng X., Su X., Jiang S., Shi R., Li Y., Lin W., Arai M. (2018). Significance of surface oxygen-containing groups and heteroatom P species in switching the selectivity of Pt/C catalyst in hydrogenation of 3-nitrostyrene. J. Catal..

[15] Li C., Shao Z., Pang M., Williams C.T., Zhang X., Liang C. (2012). Carbon nanotubes supported mono- and bimetallic Pt and Ru catalysts for selective hydrogenation of phenylacetylene. Ind. Eng. Chem. Res..

[16] Domínguez-Domínguez S., Berenguer-Murcia Á., Pradhan B.K., Linares-Solano A., Cazorla-Amorós D. (2008). Semihydrogenation of phenylacetylene catalyzed by palladium nanoparticles supported on carbon materials. J. Phys. Chem. C.

[17] Xia L., Li D., Long J., Huang F., Yang L., Guo Y., Jia Z., Xiao J., Liu H. (2019). N-doped graphene confined Pt nanoparticles for efficient semi-hydrogenation of phenylacetylene. Carbon.

[18] Wang J., Wei Q., Ma Q., Guo Z., Qin F., Ismagilov Z.R., Shen W. (2020). Constructing Co@ N-doped graphene shell catalyst via Mott-Schottky effect for selective hydrogenation of 5-hydroxylmethylfurfural. Appl. Catal. B Environ..

[19] Yadav A.A., Kang S.-W., Hunge Y.M. (2021). Photocatalytic degradation of Rhodamine B using graphitic carbon nitride photocatalyst. J. Mater. Sci. Mater. Electron..

[20] Yadav A., Hunge Y., Kang S.-W. (2021). Ultrasound assisted synthesis of highly active nanoflower-like CoMoS4 electrocatalyst for oxygen and hydrogen evolution reactions. Ultrason. Sonochem..

[21] Ahmad K., Kumar P., Mobin S.M. (2020). Hydrothermally grown novel pyramids of the CaTiO3 perovskite as an efficient electrode modifier for sensing applications. Mater. Adv..

[22] Ahmad K., Mohammad A., Mobin S.M.J.E.A. (2017). Hydrothermally grown α-MnO2 nanorods as highly efficient low cost coun-ter-electrode material for dye-sensitized solar cells and electrochemical sensing applications. Electrochim. Acta.

[23] Ahmad K., Mohammad A., Mobin S.M. (2016). Preparation of SrTiO 3 perovskite decorated rGO and electrochemical detection of nitroaromatics. Electrochimica Acta.

[24] Raza W., Ahmad K. (2018). A highly selective Fe@ ZnO modified disposable screen printed electrode based non-enzymatic glucose sensor (SPE/Fe@ ZnO). Mater. Lett..

[25] Ahmad K., Mobin S.M. (2020). Advanced functional nanomaterials for explosive sensors. Handb. Nanomater. Nanocomposites Energy Environ. Appl..

[26] Tan L., Guo X., Gao X., Tsubaki N. (2019). Designing a mesoporous zeolite catalyst for products optimizing in n-decane hydrocraking. Catalysts.

[27] Tan L., Wang F., Zhang P., Suzuki Y., Wu Y., Chen J., Yang G., Tsubaki N. (2020). Design of a core–shell catalyst: An effective strategy for suppressing side reactions in syngas for direct selective conversion to light olefins. Chem. Sci..

[28] Wang G., Sun Y., Li D., Liang H.W., Dong R., Feng X., Müllen K. (2015). Controlled synthesis of N-doped carbon nanospheres with tailored mesopores through self-assembly of colloidal silica. Angew. Chem..

[29] Xu L., Nie R., Lyu X., Wang J., Lu X. (2020). Selective hydrogenation of furfural to furfuryl alcohol without external hydrogen over N-doped carbon confined Co catalysts. Fuel Process. Technol..

[30] Xia J., He G., Zhang L., Sun X., Wang X. (2016). Hydrogenation of nitrophenols catalyzed by carbon black-supported nickel nanoparticles under mild conditions. Appl. Catal. B Environ..

[31] Han A., Chen W., Zhang S., Zhang M., Han Y., Zhang J., Ji S., Zheng L., Wang Y., Gu L. (2018). A polymer encapsulation strategy to synthesize porous nitrogen-doped carbon-nanosphere-supported metal iso-lated-single-atomic-site catalysts. Adv. Mater..

[32] Jagadeesh R.V., Murugesan K., Alshammari A.S., Neumann H., Pohl M.-M., Radnik J., Beller M. (2017). MOF-derived cobalt nanoparticles catalyze a general synthesis of amines. Science.

[33] Xiong W., Wang Z., He S., Hao F., Yang Y., Lv Y., Zhang W., Liu P., Luo H. (2020). Nitrogen-doped carbon nanotubes as a highly active metal-free catalyst for nitrobenzene hydrogenation. Appl. Catal. B Environ..

[34] Chen Y., Wang Z., Mao S., Wang Y. (2019). Rational design of hydrogenation catalysts using nitrogen-doped porous carbon. Chin. J. Catal..

[35] Wei Q., Wang J., Shen W. (2021). Atomically dispersed Feδ+ anchored on nitrogen-rich carbon for enhancing benzyl alcohol oxidation through Mott-Schottky effect. Appl. Catal. B Environ..

[36] Ruan L., Pei A., Liao J., Zeng L., Guo G., Yang K., Zhou Q., Zhao N., Zhu L., Chen B.H. (2021). Nitrogen-doped carbon nanotubes-supported PdNiCo nanoparticles as a highly efficient catalyst for selective hydro-genation of furfural. Fuel.

[37] Nie R., Peng X., Zhang H., Yu X., Lu X., Zhou D., Xia Q. (2017). Transfer hydrogenation of bio-fuel with formic acid over biomass-derived N-doped carbon supported acid-resistant Pd catalyst. Catal. Sci. Technol..

[38] Inagaki M., Toyoda M., Soneda Y., Morishita T. (2018). Nitrogen-doped carbon materials. Carbon.

[39] Yang L., Cheng D., Xu H., Zeng X., Wan X., Shui J., Xiang Z., Cao D. (2018). Unveiling the high-activity origin of single-atom iron catalysts for oxygen reduction reaction. Proc. Natl. Acad. Sci. USA.

[40] Jagadeesh R.V., Surkus A.-E., Junge H., Pohl M.-M., Radnik J., Rabeah J., Huan H., Schünemann V., Brückner A., Beller M. (2013). Nanoscale Fe_2_O_3_-based catalysts for selective hydrogenation of nitroarenes to anilines. Science.

[41] Indra A., Beltrán-Suito R., Müller M., Sivasankaran R.P., Schwarze M., Acharjya A., Pradhan B., Hofkens J., Brückner A., Thomas A. (2021). Promoting photocatalytic hydrogen evolution activity of graphitic carbon nitride with hole-transfer agents. ChemSusChem.

[42] Bhardwaj N., Singh A.K., Tripathi N., Goel B., Indra A., Jain S.K. (2021). Ni–NiO heterojunctions: A versatile nanocatalyst for regioselective halogenation and oxidative esterification of aromatics. New J. Chem..

[43] Singh B., Indra A. (2021). Tuning the properties of CoFe-layered double hydroxide by vanadium substitution for improved water splitting activity. Dalton Trans..

[44] Indra A., Acharjya A., Menezes P.W., Merschjann C., Hollmann D., Schwarze M., Aktas M., Friedrich A., Lochbrunner S., Thomas A. (2017). Boosting visible-light-driven photocatalytic hydrogen evolution with an integrated nickel phosphide-carbon nitride system. Angew. Chem..

[45] Dutta S., Indra A., Han H., Song T. (2018). An intriguing pea-like nanostructure of cobalt phosphide on molybdenum carbide incorporated nitrogen-doped carbon nanosheets for efficient electrochemical water splitting. ChemSusChem.

[46] Ma X., Zhou Y.X., Liu H., Li Y., Jiang H.L. (2016). A MOF-derived Co–CoO@ N-doped porous carbon for efficient tandem catalysis: Dehydrogenation of ammonia borane and hydrogenation of nitro compounds. Communications.

[47] Wei Z., Wang J., Mao S., Su D., Jin H., Wang Y., Xu F., Li H., Wang Y. (2015). In situ-generated Co^0^-Co_3_O_4_/N-doped carbon nanotubes hybrids as efficient and chemoselective catalysts for hydro-genation of nitroarenes. ACS Catal..

[48] Duan B., Gao X., Yao X., Fang Y., Huang L., Zhou J., Zhang L. (2016). Unique elastic N-doped carbon nanofibrous microspheres with hierarchical porosity derived from renewable chitin for high rate supercapacitors. Nano Energy.

[49] Yang Y., He F., Shen Y., Chen X., Mei H., Liu S., Zhang Y. (2017). A biomass derived N/C-catalyst for the electrochemical production of hydrogen peroxide. Chem. Commun..

[50] Su H., Zhang K.-X., Zhang B., Wang H.-H., Yu Q.-Y., Li X.-H., Antonietti M., Chen J.-S. (2017). Activating cobalt nanoparticles via the Mott–Schottky effect in nitrogen-rich carbon shells for base-free aerobic oxidation of alcohols to esters. J. Am. Chem. Soc..

[51] Liu W., Zhang L., Liu X., Liu X., Yang X., Miao S., Wang W., Wang A., Zhang T. (2017). Discriminating catalytically active FeN_x_ species of atomically dispersed Fe–N–C catalyst for selective oxidation of the C–H bond. J. Am. Chem. Soc..

[52] Jia J., Zhang P., Chen L. (2016). Catalytic decomposition of gaseous ozone over manganese dioxides with different crystal structures. Appl. Catal. B Environ..

[53] Chen T., Guo S., Yang J., Xu Y., Sun J., Wei D., Chen Z., Zhao B., Ding W. (2017). Nitrogen-doped carbon activated in situ by embedded nickel through the Mott-Schottky effect for the oxygen reduction reaction. ChemPhysChem.

[54] Men Y., Tan Y., Li P., Cao X., Jia S., Wang J., Chen S., Luo W. (2021). Tailoring the 3d-orbital electron filling degree of metal center to boost alkaline hydrogen evolution electrocatalysis. Appl. Catal. B Environ..

[55] Li X., Pan Y., Yi H., Hu J., Yang D., Lv F., Li W., Zhou J., Wu X., Lei A. (2019). Mott–Schottky effect leads to alkynes semihydrogenation over Pd-nanocube@ N-doped carbon. ACS Catal..

[56] Sun Z., James D.K., Tour J.M. (2011). Graphene chemistry: Synthesis and manipulation. J. Phys. Chem. Lett..

[57] Seo D.-H., Lee J., Urban A., Malik R., Kang S., Ceder G. (2016). The structural and chemical origin of the oxygen redox activity in layered and cation-disordered Li-excess cathode materials. Nat. Chem..

[58] Li J., Liu G., Long X., Gao G., Wu J., Li F. (2017). Different active sites in a bifunctional Co@ N-doped graphene shells based catalyst for the oxidative dehydrogenation and hydrogenation reactions. J. Catal..

[59] Deng D., Yu L., Chen X., Wang G., Jin L., Pan X., Deng J., Sun G., Bao X. (2013). Iron encapsulated within pod-like carbon nanotubes for oxygen reduction reaction. Angew. Chem..

[60] Li X.-H., Antonietti M. (2013). Metal nanoparticles at mesoporous N-doped carbons and carbon nitrides: Functional Mott–Schottky heterojunctions for catalysis. Chem. Soc. Rev..

